# A recommendation model based on profile enhancement for personalized medical expert in online health communities

**DOI:** 10.3389/fpubh.2026.1882351

**Published:** 2026-09-28

**Authors:** Yan Shen, Hui Long, Qian Wang

**Affiliations:** School of Management, Jiangsu University, Jiangsu, China

**Keywords:** online health communities, personalized recommendation, platform governance, profiling, value co-creation

## Abstract

**Introduction:**

Inefficient matching between medical experts and health advice seekers remains a challenge in online health communities. This study constructs the Recommendation Model with Profile Enhancement (RMPE), which integrates service quality and user sentiment dimensions to enhance service exchange efficiency, promote value co-creation, and provide algorithmic support and managerial insights for the ecological governance of online health communities.

**Methods:**

The RMPE workflow consists of three stages: extracting dynamic expert profiles via topic modeling, quantifying users’ subjective experiences through sentiment analysis, and completing personalized recommendations via collaborative filtering. The model computationally integrates Service-Dominant Logic and Information Ecology Theory into the recommendation process.

**Results:**

Empirical analysis based on data from Haodf.com demonstrates that RMPE achieves a recommendation accuracy of 88.1%, significantly outperforming traditional baseline models, and INTERNAL/BUSINESS successfully identifies over 80% of users’ final choices within the Top-30 recommendation list.

**Discussion:**

The findings validate the technical effectiveness of the profile enhancement pathway and provide a framework for quantitatively analysing value co-creation processes in online health communities. The proposed approach provides both Theoretical and practical value for platform governance by supporting resource allocation optimization, community culture cultivation, and the sustainable development of the health community ecosystem.

## 1 Introduction

“Digital Health” has emerged as a significant driver for the rapid development of the health information industry in the new era. According to the 55th Statistical Report on the Development Status of the Internet in China (1), by December 2025, the number of internet medical service users in China had reached 418 million, accounting for 37.7% of all internet users. Internet medical services have achieved rapid development.

The massive health-related data accumulated by online health communities constitutes a valuable information resource. However, health advice seekers, who often lack professional medical knowledge, frequently struggle to select appropriate medical experts to address their health issues due to information overload (2). Consequently, enhancing the utilization of online medical information and achieving personalized matching between experts and advice seekers have remained a key research focus within this field.

Existing research primarily focuses on the similarity of question-answer texts. However, relying solely on textual similarity fails to accurately reflect the professional competence of medical experts. Profiling technology, which extracts key individual information from massive datasets, is now widely applied in areas such as targeted marketing, product recommendation, and decision support. At present, data such as experts’ self-descriptions, research experience, and health advice seekers’ evaluations in online health communities have gradually grown in scale, providing important support for constructing medical expert profiles. Nevertheless, from the perspective of internet platform governance, such a matching mechanism is significant not only for improving individual user experience but also for interpreting experts’ value propositions and advice seekers’ value perceptions. It is crucial for optimizing the platform’s core resource allocation, fostering community value co-creation, and maintaining the sustainable development of the online health community ecosystem. To address this deeper managerial need, this study proposes a precision matching pathway based on profile enhancement. It aims to integrate and analyze multi-source interaction data, providing core algorithmic support at the level of optimizing the information ecology to improve community service quality and promote its sustainable development.

## 2 Literature review

As a critical platform connecting medical experts and health advice seekers, the core function of Online Health Communities (OHCs) lies in facilitating information exchange and resource matching. To address how to enhance platform service efficiency and user satisfaction, existing research has been conducted from multiple perspectives, including community structure, user behavior, profile construction, and intelligent recommendation systems.

Early research focused on the systematic deconstruction of online health communities and the analysis of user behavior. Zhao Dongxiang (3) pointed out that OHCs constitute a complex information ecosystem composed of community, users, and information. Xi Haitao et al. (4), through bibliometric analysis, further revealed patterns of user interaction within these communities and their significance for constructing a medical ecosystem. Subsequent studies delved deeper into user behavior patterns. For instance, Zhang Jun et al. (5), based on knowledge co-construction theory, explored the patterns of users’ knowledge-sharing behavior by constructing user interaction behavior networks and knowledge networks. Ouyang et al. (6) empirically investigated how physicians’ free knowledge-sharing influences their popularity and attractiveness to health advice seekers. Zou Xiao et al. (7) proposed a group decision analysis framework for recommending optimal expert responses, integrating text analysis and complex network analysis based on evidence theory. Furthermore, scholars have employed methods such as knowledge graphs (8), evolutionary game theory (9), and social network analysis (10) to study the formation mechanisms and dissemination and sharing pathways of health information within virtual communities. These studies contribute to improving the quality of information services and user experience on community platforms, thereby promoting the healthy dissemination of health knowledge.

With the continuous expansion of community data, researchers have begun to seek more refined methods for user understanding and representation, giving rise to user profiling technology. This technology constructs user feature models by integrating multi-source data and has proven highly valuable in fields such as precision marketing and personalized recommendation. Mao Taitian et al. (11) investigated user profiles related to health information avoidance behavior on mobile social media. Yang Lei and Li Jingjing (12) proposed a user profiling construction method and an emotion propagation model that incorporate emotional features based on the structure of social network graphs, thereby optimizing the emotional support function of online health communities. Zhang Yanfeng et al. (13) constructed profiles of health-anxious users in online medical communities to reveal the demand and behavioral characteristics of different types of health-anxious user groups, providing a basis for achieving precise health information recommendation and personalized services. Early profiling studies often relied on traditional topic models like LDA for extracting group feature labels. While achieving certain results, these approaches exhibit limitations in the nuanced expression of topics and the depth of semantic understanding.

In recent years, breakthroughs in artificial intelligence, particularly in deep learning and pre-trained large language models, have provided more powerful tools for thematic analysis of health information. Guo Yu et al. (14) used the BERTopic topic model to construct topic documents and analyze the thematic trends of health information on the Bilibili social platform. Wang Dandan and Wu Jinge (15) employed LDA topic modeling, content analysis, and the PMC index model to conduct multi-dimensional mining and systematic analysis of policy texts on China’s health big data policies. Gao Chunling et al. (16) used the BERTopic model to achieve topic mining of older adult health information needs, providing a basis for medical institutions and relevant government departments to carry out health information services. The research by Yan Weiwei et al. (17) demonstrated the effectiveness of BERTopic in improving topic quality. These studies lay the foundation for extracting medical experts’ service characteristics from massive health information.

However, topic mining that focuses solely on objective characteristics remains insufficient for achieving effective personalized recommendations. User decision-making, especially in the medical and health domain, is profoundly influenced by subjective perceptions and emotional tendencies. Consequently, sentiment analysis has become a critical component in enhancing the performance of recommendation systems. The online health community recommendation method based on adaptive matrix factorization proposed by Yang and Gao (18) provides an effective solution for precision matching. The hybrid recommendation system incorporating sentiment analysis proposed by Elahi et al. (19) significantly improved recommendation effectiveness. Fan et al. (20), considering health advice seekers’ needs, utilized machine learning algorithms and sentiment analysis tools to deeply mine health advice seeker service demands and experts service capabilities, constructing a multi-stage dynamic experts-health advice seeker matching model to enhance the accuracy and diversity of experts recommendations. Li et al. (21) measured health advice seeker satisfaction based on emotional information contained in health advice seeker review texts and then combined it with TOPSIS to derive experts’ rankings. The aforementioned studies collectively substantiate that integrating sentiment analysis technology can significantly improve the accuracy and diversity of recommendation outcomes.

Existing research provides a solid foundation for this study. However, from the perspective of platform governance and ecological optimization, current studies still exhibit limitations in supporting the sustainable development of online health communities. On the one hand, when characterizing medical experts-the core platform asset-existing profiles often focus on static attributes or single textual topics, failing to deeply quantify the multidimensional value propositions, such as professionalism and empathy, embedded in their service interactions. On the other hand, regarding the recommendation mechanism, further in-depth research is warranted in how to systematically integrate experts’ dynamic service profiles with health advice seekers’ subjective emotional feedback to model and promote value co-creation within the platform. Importantly, recent empirical work (22) has demonstrated that the core tenets of Service-Dominant Logic, particularly the value co-creation process, are amenable to quantitative operationalization in collaborative settings. This lends direct support to our endeavor, suggesting that a computable framework-rooted in enhanced SERVQUAL scoring and user sentiment mining-can effectively capture the multidimensional service value embedded in expert-seeker interactions within online health communities.

This study constructs a medical expert profile by integrating BERTopic with the improved SERVQUAL model. Based on this expert profile, the study further combines fine-grained sentiment analysis of health advice seekers with deep learning to optimize the collaborative filtering recommendation model, aiming to achieve more accurate matching between experts and advice seekers.

## 3 Research framework and methodology

### 3.1 Research framework

This study is grounded in two core theories: Service Dominant Logic and Information Ecology Theory. It aims to construct a personalized medical expert recommendation system designed to serve value co-creation and ecological balance within online health communities.

First, grounded in Service-Dominant Logic, this study conceptualizes medical and health services as a process of co-creating value between medical experts and health advice seekers. The professional diagnosis, emotional care, and other services provided by medical experts, together with the consultations, review feedback, and other contributions from health advice seekers, form integrated resources through interaction and jointly determine service value. Specifically, the operant resources emphasized in Service-Dominant Logic, such as medical experts’ professional knowledge, clinical experience, and empathy, are systematically identified and quantified through BERTopic topic modeling and the SERVQUAL framework. These operant resources are systematically transformed into structured profile scores covering five dimensions, including professionalism, empathy, reliability, assurance, and responsiveness. In this way, the operant resources are represented in a computable form. Traditional recommendation systems often focus on the static attributes of medical experts. They fail to fully reflect the dynamic process of value co-creation. Therefore, this study redefines the medical expert profile as a dynamic representation of the expert’s service capabilities and value propositions. The five SERVQUAL dimensions-professionalism, empathy, reliability, assurance, and responsiveness-are mapped to the key service elements within value co-creation. The medical expert profile score is thus defined as a quantitative expression of the expert’s value proposition. Concurrently, the historical behavior and emotional feedback of health advice seekers are viewed as records of their participation in value co-creation. The core function of the recommendation model is to improve the efficiency of value co-creation between experts and health advice seekers. It achieves this by accurately matching the value propositions of medical experts with the participation patterns of health advice seekers. In other words, value co-creation is operationalized in this study as the collaborative filtering matching process between the profile score and the sentiment label. When the emotional feedback of a health advice seeker matches the service profile of a medical expert, a successful value co-creation event is formed.

Second, drawing upon Information Ecology Theory, this study conceptualizes the online health community as an interdependent ecosystem composed of information, information actors, and the information environment. In this study, information refers to medical expert profile features and the personalized needs of health advice seekers. It includes computable structured data, such as the profile score, the sentiment label, and the BERT feature vector. Information actors include medical experts who provide services, health advice seekers who seek services and generate feedback, and platform managers who operate and optimize the platform. The information environment refers to the multi-sided platform of the online health community. Therefore, this study does not treat the recommendation algorithm as an isolated technical tool. Instead, it situates the algorithm within the ecosystem formed by medical experts, health advice seekers, and the online health community. It also pays attention to the interactions among these elements. The contribution of the RMPE model to the information ecology lies in improving the efficiency of information flow among these three elements. Through profile enhancement, information, namely experts’ service features, can be more accurately matched with information actors, namely health advice seekers’ needs. This allows more efficient resource allocation within the information environment, namely the platform. In essence, the recommendation algorithm optimizes the efficiency of information flow within the ecosystem. This is the core way in which this study computationalizes Information Ecology Theory.

Based on this logic, this study constructs a framework characterized as “Data-Driven-Profile Enhancement-Value Matching-Recommendation Generation” as illustrated in Figure 1. This framework embodies the logic of extracting value co-creation elements from data to achieve profile enhancement, followed by facilitating value exchange within the information ecology to accomplish value matching.

**Figure 1:**
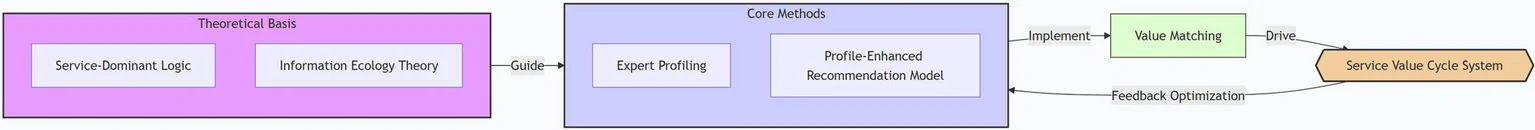
Research framework.

Based on the aforementioned research ideas, the core objective of this study is to construct a recommendation model that serves the platform governance of Online Health Communities. This requires the model to not only achieve precise matching but also function as a data-driven governance tool. It should quantify service value and uncover user sentiment to optimize the matching mechanism, thereby serving the health of the platform ecosystem. To accomplish this goal, this study designs and implements the Recommendation Model with Profile Enhancement (RMPE). Specifically, the process involves three key steps. First, BERTopic topic modeling and an enhanced SERVQUAL framework are used to construct medical expert profiles that reflect multidimensional service value from interaction data. Second, a Residual Connection-based LSTM model is employed to perform fine-grained sentiment analysis on user reviews, quantifying their subjective experiences. Finally, enhanced expert profiles and user sentiment feedback are deeply integrated into a collaborative filtering recommendation framework to build the RMPE model, enabling personalized recommendation within the online health community. Within this collaborative filtering stage, a mean-centered cosine similarity is adopted to measure user preference similarity, effectively mitigating the bias caused by individual differences in rating scales.

### 3.2 Research methodology

#### 3.2.1 Construction of medical expert profiles in online health communities

The RMPE model constructed in this section involves multiple variables. To facilitate the expression of the subsequent formulations, the key variables and their definitions are summarized in Table 1.

**Table 1:** Key variables and definitions in the RMPE model.

Symbol	Definition	Role in the model
*P_j_*	Integrated sentiment score of medical expert *j*	Adjusts SERVQUAL feature weights
*F_j_*	Deep semantic representation of medical expert *j*	Integrated with SERVQUAL features and contributes to the final profile score
MLP(*F_j_*)	Multi-layer perceptron that maps *F_j_* to a scalar semantic score	Maps *F_j_* to a scalar score
*β*	Fusion weight hyperparameter	Balances structured profile and semantic scores
$w_{k}^{\mathrm{new}}$	Adjusted weight of the *k-th* SERVQUAL dimension	Used in the final profile score computation
*γ*	Feature-weight adjustment rate	Controls the magnitude of weight updates
*S_j_*	Final profile score of medical expert *j*	Represents the enhanced expert profile
*α*	Hyperparameter balancing historical ratings and profile scores	Balances historical ratings and profile scores
sim(*u_i_,u_j_*)	Mean-centered cosine similarity between health advice seekers *u_i_* and *u_j_*	Measures preference similarity in collaborative filtering
${\hat{r}}_{\mathrm{ud}}$	Predicted rating of health advice seeker *u* for medical expert *d*	Determines the final recommendation ranking

The construction of precise medical expert profiles mainly involves the analysis of basic characteristics and service characteristics. First, the review data from health advice seekers are pre-processed, which includes data cleaning, tokenization using the “jieba” library, removal of stop words, and creation of a dictionary to ensure textual data standardization. Subsequently, the BERTopic topic model is employed to identify the main topics within the reviews. Multidimensional Scaling (MDS) analysis and K-means clustering are applied for dimensionality reduction and cluster analysis of the data. To extract service characteristics, this study refers to previous studies (23–25) and uses an improved SERVQUAL model to classify the medical expert profile according to five dimensions, including professionalism, empathy, reliability, assurance, and responsiveness. Finally, the BERTopic model is applied to extract expert features, generating a precise profile for each medical expert. The complete workflow is illustrated in Figure 2.

**Figure 2:**
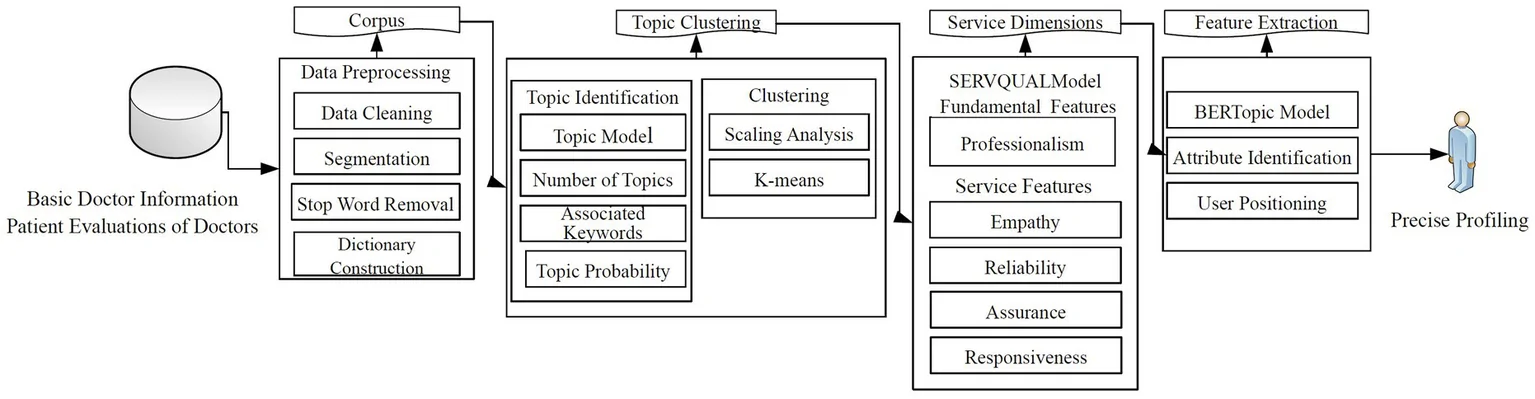
Medical expert precise portrait construction process.

##### 3.2.1.1 Construction of the topic model based on BERTopic

First, the BERTopic model is employed to transform medical experts’ self-descriptions, historical consultations, and post-consultation reviews into high-dimensional semantic vectors that preserve contextual information. The generation process is described as follows:


X=SBERT(T)

Here, *X* represents the embedding vector of the text, *T* denotes the input text, and *SBERT* refers to the Sentence-BERT pre-trained encoder. Through text embedding, each textual instance is transformed into a 768-dimensional vector that encapsulates semantic information.

Next, topic generation and quantity determination are conducted. The BERTopic model defaults to using Uniform Manifold Approximation and Projection (UMAP) for dimensionality reduction, which can preserve the topological structure of the original high-dimensional data in a lower-dimensional space:


Y=UMAP(X)

Subsequently, the K-means algorithm is applied to cluster the dimensionally reduced text vectors. Let *k* denote the number of clusters, *S_i_* represent the *i-th* cluster, and *μ_i_* be the centroid of that cluster. The objective function of K-means is defined as follows:


$$J=\sum_{i=1}^{k}\sum_{x_{j}\in S_{i}}∥x_{j}-\mu_{i}{∥}^{2}$$

Based on the clustering results, BERTopic extracts the keywords corresponding to each topic. In this study, keyword extraction is performed using the Term Frequency-Inverse Document Frequency (TF–IDF) algorithm. Let *t* denote a term, *d* represent a document, *N* be the total number of documents, and *DF(t)* denote the number of documents containing the term *t*. The TF–IDF keyword extraction process is defined as follows:


$$\mathrm{TF}-\mathrm{IDF}(t,d)=\mathrm{TF}(t,d)\times \log(\frac{N}{\mathrm{DF}(t)})$$

Based on the TF–IDF results, the keywords of each topic are extracted to ensure that every topic is described by a set of most representative terms that capture its underlying meaning. After topic generation, BERTopic calculates the probability that each text corresponds to each topic.

##### 3.2.1.2 Generation of precise medical expert profiles

To provide a structured, multi-perspective description of medical expert services, this study adopts an improved SERVQUAL model as the theoretical framework for constructing medical expert service profiles. Although the SERVQUAL model originated in traditional service industries, its five core dimensions-Reliability, Assurance, Tangibles, Empathy, and Responsiveness-provide a universal foundation for measuring service quality. This model has been referenced and applied by several scholars in the field of health information, particularly in the evaluation of online health services (24, 25), demonstrating its good extensibility within the context of health information consultation.

However, the “Tangibles” dimension in the classic SERVQUAL model refers to physical facilities and equipment. In the online virtual environment, this dimension becomes less relevant to medical expert services. Therefore, this study makes a key adaptation by removing the “Tangibles” dimension and simultaneously introducing and highlighting the “Professionalism” dimension as a foundational feature of the medical expert profile. This adjustment better aligns with the core characteristic of online health communities, where “information and service” are paramount.

To describe the service performance of medical experts from multiple perspectives, this study employs an improved SERVQUAL model comprising the five dimensions of Professionalism, Empathy, Reliability, Assurance, and Responsiveness.

1) Professionalism as the Foundational Characteristic Dimension. This dimension is primarily reflected in the medical expert’s academic background, medical education history, technical proficiency, and clinical competencies demonstrated in actual diagnosis and treatment processes.
2) Service Characteristic Dimensions. These dimensions consist of Empathy, Reliability, Assurance, and Responsiveness. Empathy refers to the care and understanding demonstrated by medical experts during the diagnosis and treatment process. Reliability primarily measures the trustworthiness and dependability of the medical expert in their diagnosis and treatment. Assurance denotes the sense of safety and trust instilled in the health advice seeker by the medical expert. Responsiveness measures the speed and proactiveness with which the medical expert reacts to the advice seeker’s needs and offers assistance.

In this study, BERTopic was used to extract topics from review texts. Three researchers independently assigned each topic to one of the five SERVQUAL dimensions based on the topic keywords. They resolved disagreements through discussion and reached a final consensus mapping. The Krippendorff’s Alpha coefficient was 0.87, indicating a high level of inter-rater agreement. The specific classification results are shown in Table 2. Through this categorization process, the service quality and professionalism of medical experts can be evaluated in a comprehensive and structured manner. This process also helps generate accurate medical expert profiles that are both theoretically grounded and adapted to the online health community context.

**Table 2:** The themes encompassed by each dimension of SERVQUAL.

Dimension	Topic no.	Topic name
Empathy	1	Medical Expert-Health advice seeker Communication
	2	Health advice seeker Emotional Experience
	3	Expert’s Caring Attitude and Health advice seeker Appreciation
	4	Emotional Resonance Between Expert and Health advice seeker
	5	Personalized Service and Health advice seeker Psychological Care
	6	Overall Service Experience and Health advice seeker Subjective Feedback
Reliability	7	The Consistency and Stability of the Consultation Effect
	8	Expert’s Sense of Responsibility and Fulfillment of Commitments
	9	Reliability of the Service Process and Health advice seeker’s Sense of Security
	10	Long-term Effectiveness and Health Advice Seeker’s Sustained Trust
Assurance	11	Expert’s Professional Authority and Health advice seeker’s Sense of Trust
	12	Expert’s Rigorous Attitude and Health advice seeker’s Sense of Safety
	13	Expert’s Reputation and Reliable Image
	14	Expert’s Educational Background and Qualification Credentials
Responsiveness	15	Convenience and Timeliness of Online Services
	16	Expert’s Rapid Response to Health advice seeker Needs
Professionalism	17	Technical Proficiency and Effectiveness in Health Information Consultation
	18	Expert’s Professional Qualities and Dedication
	19	Practical Experience and Ability to Handle Complex Cases
	20	Knowledge of Health Service Theory and Research Capability
	21	Systematic Health Education and Professional Training

#### 3.2.2 Construction of the RMPE recommendation model

Building upon the precise medical expert profiles, this section integrates sentiment analysis of health advice seeker reviews to design the RMPE model-a collaborative filtering-based personalized medical expert recommendation model enhanced by profiling. The overall framework is illustrated in Figure 3.

**Figure 3:**
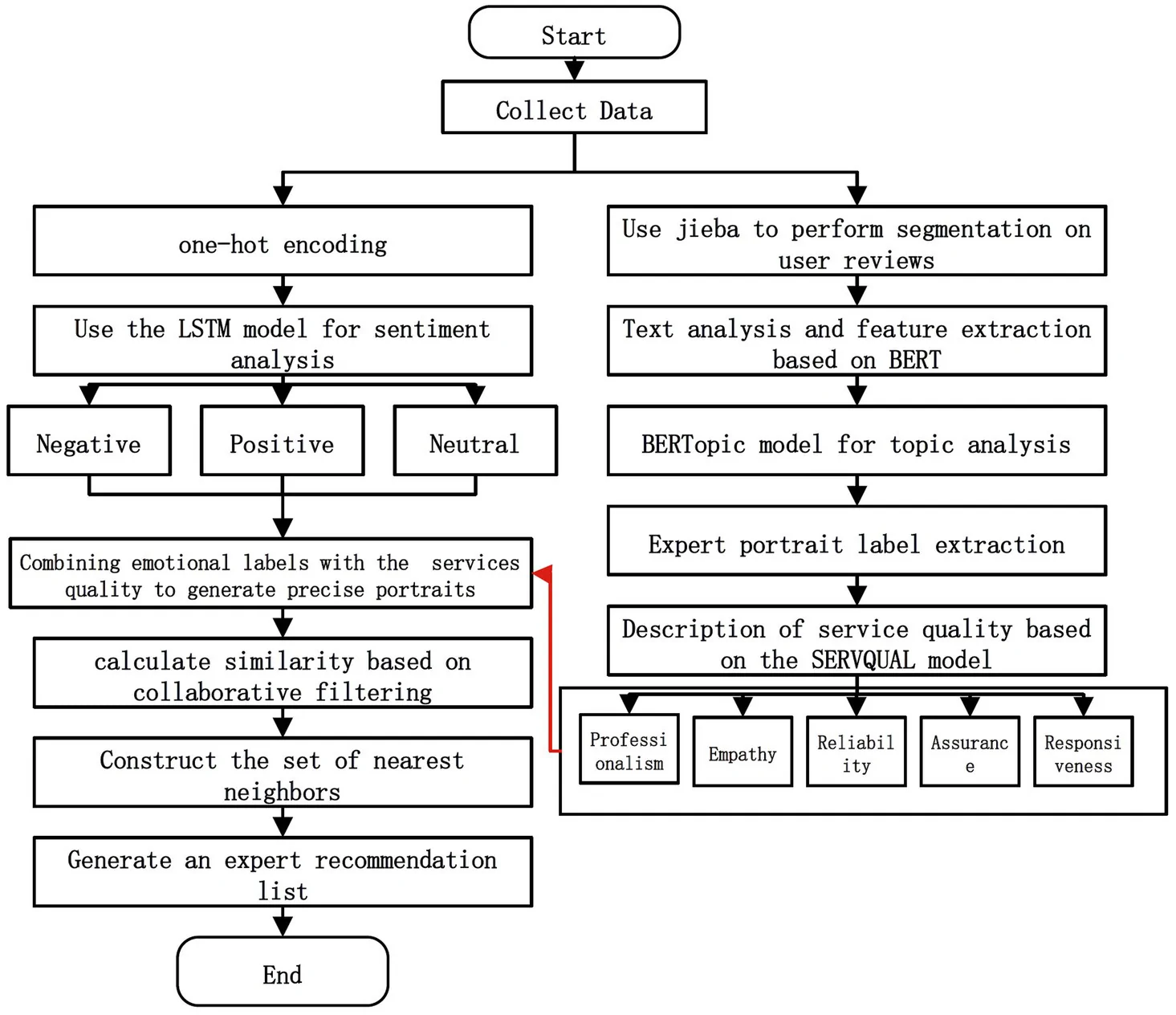
Medical expert recommendation model RMPE.

##### 3.2.2.1 Sentiment analysis based on residual-connected LSTM

To quantify the subjective experiences of health advice seekers from their reviews, this module generates a sentiment label for each review through sentiment analysis. This study employs an LSTM model based on residual connections. By incorporating residual connection layers, the model captures complex emotional features in review texts and provides sentiment labels for personalized medical expert recommendation.

First, one-hot encoding is utilized to represent words in the text as high-dimensional sparse vectors, assigning a unique vector code to each word. The frequency of all words in the review data is counted and duplicates are removed, forming the vocabulary *V*, which is the set of all unique words with a size of *|V|*. For a word *w* in vocabulary *V*, whose position is *i*, its one-hot encoding vector *e_w_* is defined such that the *i-th* element is 1 and all other elements are 0. The one-hot encoding vector *e_w_* for word *w* can be defined as follows:


$$e_{w}[j]=\{\begin{array}{c} 1,\text{if}\;j=\text{index}(w)\\ 0,\text{otherwise} \end{array}$$

Subsequently, sentiment analysis is conducted using an LSTM model based on residual connections, as illustrated in Figure 4. When processing extremely long sequences, traditional LSTM models may still encounter gradient vanishing or gradient explosion (26). By introducing residual connections, neural networks can effectively mitigate the gradient vanishing problem and enhance the model’s sensitivity to subtle emotional changes in review sentences.

**Figure 4:**
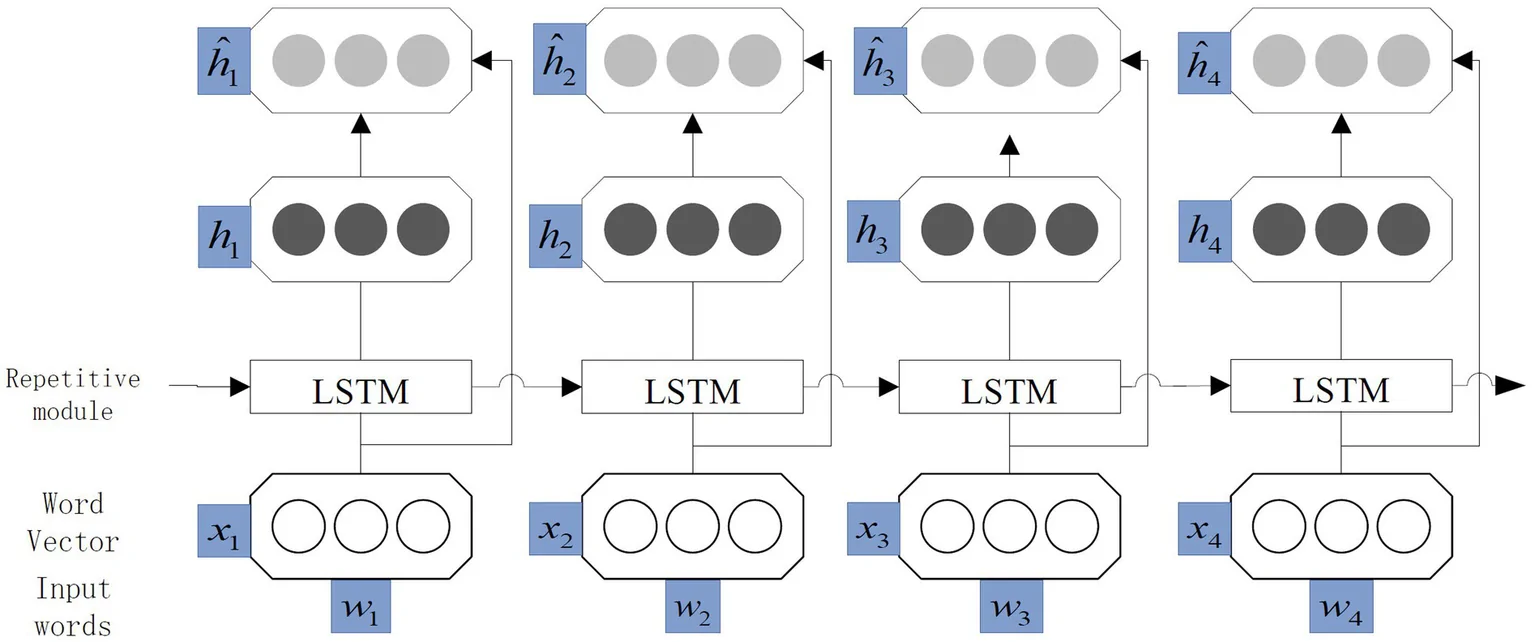
LSTM network structure based on residual connections.

The results of the one-hot encoding are then fed into the LSTM layer. The LSTM progressively updates its hidden state to retain important emotional information from the review. For each timestep *t*, the core computational process of the LSTM is as follows:


$$f_{t}=\sigma (W_{f}\cdot [h_{t-1},x_{t}]+b_{f})$$

$$i_{t}=\sigma (W_{i}\cdot [h_{t-1},x_{t}]+b_{i})$$

$${\tilde{C}}_{t}=\tan h(W_{C}\cdot [h_{t-1},x_{t}]+b_{C})$$

$$C_{t}=f_{t}*C_{t-1}+i_{t}*{\tilde{C}}_{t}$$

$$o_{t}=\sigma (W_{o}\cdot [h_{t-1},x_{t}]+b_{o})$$

$$h_{t}=o_{t}*\tan h(C_{t})$$

Here, *f_t_* denotes the forget gate, *i_t_* represents the input gate, and ${\tilde{C}}_{t}$ is the candidate cell state, *C_t_* denotes the current cell state, *O_t_* is the output gate, and *h_t_* represents the current hidden state. *W_f_*, *W_i_*, *W_C_*, and *W_O_* are the weight matrices of the LSTM, while *b_f_*, *b_i_*, *b_C_*, and *b_o_* denote the bias terms. σ denotes the sigmoid activation function. Based on the traditional LSTM, residual connections are introduced as follows:


$$h_{t}^{\mathrm{res}}=h_{t}+Wx_{t}$$

Here, *W* denotes the linear transformation matrix, which is used to adjust *x_t_* to have the same dimensionality as *h_t_*. The residual output at each time step, denoted as *h_t_^res^*, is then fed into a fully connected layer, where a *softmax* activation function is applied to obtain the final sentiment classification result. Let the output sentiment category be represented as *y*, which can be defined as follows:


$$y=\text{softmax}(W_{y}\cdot h_{n}^{\mathrm{res}}+b_{y})$$

Here, *h_t = n_^res^* represents the residual connection output of the final time step in the LSTM. *W_y_* and *b_y_* denote the weight matrix and bias term of the fully connected layer, respectively. During training, the model parameters are optimized using the cross-entropy loss function. Let *k* denote the number of sentiment categories, *y_i_* be the true label, and ${\hat{y}}_{i}$ represent the predicted probability produced by the model. The loss function is defined as follows:


$$L=-\sum_{i=1}^{k}y_{i}\log({\hat{y}}_{i})$$

##### 3.2.2.2 Feature learning based on BERT

This study employs a pre-trained BERT model to extract effective features from health advice seeker reviews for profile-enhanced recommendation. Each review *d_i_* is processed through an embedding layer, and the vectorized sequence is then input into the Transformer architecture of BERT:


$$H^{\left(l\right)}=\text{Transformer}(H^{\left(l-1\right)})$$

Here, *H*^(0)^ represents the initial input generated by the embedding layer, while *H*^(l)^ denotes the output representation of the l-th layer. Through layer-by-layer feature extraction and information interaction, the final output of the L-th layer, *H*^(L)^ is obtained, which contains the deep feature representation of the text. In the output layer of BERT, the first token vector, *H*^(L)^_[CLS]_, serves as the overall semantic representation of the review text.


$$R_{i}=H_{\left[\mathrm{CLS}\right]}^{\left(L\right)}$$

Each review *d_i_* is transformed into a fixed-length vector *R_i_*, representing the feature representation of that review. For a given expert *j*, all review feature vectors are collected to form the feature set *D_j_ = {R_1_, R_2_, …, R_|Dj|_}*, which is then aggregated to obtain the expert’s overall feature representation:


$$F_{j}=\frac{1}{|D_{j}|}\sum_{i=1}^{|D_{j}|}R_{i}$$

Through BERT embedding and feature extraction, the deep semantic representation *F_j_* of a medical expert can be constructed. This vector represents the aggregated semantic information of review texts. It is integrated with the five SERVQUAL-based profile features *F = {f1, f2,…, f5}*. The fusion weight is denoted as *β*, and its optimal value is determined through hyperparameter tuning. *F_j_* is mapped into a semantic score through an MLP and then jointly contributes to the final expert profile score. This process enables a multi-level representation of medical experts, from shallow statistical features to deep semantic features.

##### 3.2.2.3 Weighted scoring of medical expert profiles

For each review *d_i_* provided by a health advice seeker regarding a medical expert, it is mapped to a corresponding sentiment label *y_i_*, forming a sentiment label set *Y = {y_1_, y_2_,…,y_N_}*, where *N* is the total number of reviews. The medical expert profile feature set is *F = {f_1_, f_2_, f_3_, f_4_, f_5_}*, which corresponds to the five dimensions of Professionalism, Empathy, Reliability, Assurance, and Responsiveness. To reflect the comprehensive multidimensional representation of the medical expert in the health advice seeker reviews, the integrated sentiment score *P_j_* for the expert is calculated as follows:


$$P_{j}=\frac{1}{|D_{j}|}\sum_{i=1}^{|D_{j}|}y_{i}$$

The sentiment scores are utilized to adjust the feature weights and the final score of each expert. Let the initial feature weight be denoted as *w_k_^old^*; the updated feature weight can then be adjusted as follows:


$$w_{k}^{\mathrm{new}}=w_{k}^{\mathrm{old}}+\gamma \cdot P_{j}\cdot f_{k}$$

Here, *γ* represents the adjustment rate, which controls the magnitude of the weight update.

Based on the adjusted feature weights, the final profile score of expert *j*, denoted as *S_j_*, is computed as a weighted combination of the SERVQUAL-based structured profile features and the BERT-derived deep semantic representation. This design ensures that both interpretable service quality dimensions and implicit semantic signals from review texts are jointly considered. The formulation is as follows:


$$S_{j}=\sum_{k=1}^{5}w_{k}^{\mathrm{new}}*f_{k}+\beta *\mathrm{MLP}(F_{j})$$

Here, *w_k_^new^* denotes the adjusted feature weight, and *f_k_* represents the expert’s performance in the *k-th* feature dimension. MLP ( $F_{j}$ ) denotes a multilayer perceptron that maps the 768-dimensional semantic vector into a scalar score. *β* denotes a hyperparameter used to balance the SERVQUAL based structured profile score and the BERT based semantic score, whose optimal value is determined via grid search on the validation set in the subsequent experimental stage.

##### 3.2.2.4 Profile-enhanced collaborative filtering recommendation

Following the preceding steps, the precise profile scores of medical experts and the sentiment orientation of health advice seekers have been obtained. This section integrates this information with traditional collaborative filtering to generate the final personalized recommendations.

First, data on the behavior of health advice seekers is collected. Let the set of health advice seekers be *U = {u_1_, u_2_, …, u_n_}* and the set of medical experts be *D = {d_1_, d_2_, …, d_m_}*. A “health advice seeker-expert” rating matrix *R* is constructed. An element *r_ud_* in the matrix represents the rating given by health advice seeker *u* to medical expert *d*. If an advice seeker has not rated a particular expert, the corresponding value is null. An example of the rating matrix is as follows:


$$R=(\begin{array}{ccc} r_{11} & r_{12} & r_{13}\\ r_{21} & r_{22} & r_{23}\\ r_{31} & r_{32} & r_{33} \end{array})$$

Next, this study used the mean-centered cosine similarity to calculate the similarity between health advice seekers. The equation essentially calculates cosine similarity on mean-centered rating vectors. This transformation centers each user’s rating distribution at zero, effectively removing user-specific offset effects while preserving the relative preference ordering. When the similarity is computed based on the set of medical experts commonly rated by two health advice seekers, this formula is mathematically equivalent to the Pearson correlation coefficient (27, 28). Therefore, compared with the original cosine similarity, the mean-centered similarity calculation can effectively reduce the bias caused by differences in user rating scales and can more accurately characterize preference similarity among health advice seekers. The similarity between health advice seekers *u*_i_ and *u_j_* is calculated as follows:


$$\mathrm{sim}(u_{i},u_{j})=\frac{\sum_{d\in D}(r_{\mathrm{id}}-\bar{r_{i}})*(r_{\mathrm{jd}}-\bar{r_{j}})}{\sqrt{\sum_{d\in D}{\left(r_{\mathrm{id}}-\bar{r_{i}}\right)}^{2}}*\sqrt{\sum_{d\in D}{\left(r_{\mathrm{jd}}-\bar{r_{j}}\right)}^{2}}}$$

The ratings that a target advice seeker might give to medical experts are predicted by calculating a weighted average of the ratings from other advice seekers, weighted by their similarity to the target advice seeker. Let *N(u)* denote the set of advice seekers similar to health advice seeker *u*. The predicted rating for a medical expert ${\hat{r}}_{\mathrm{ud}}$ is then given by:


$${\hat{r}}_{\mathrm{ud}}=\frac{\sum_{v\in N(u)}\mathrm{sim}(u,v)\cdot r_{\mathrm{vd}}}{\sum_{v\in N(u)}|\mathrm{sim}(u,v)|}$$

For medical expert *d_j_*, incorporating their precise profile score *S_j_* into the recommendation model, the aforementioned formula can be adjusted as follows:


$${\hat{r}}_{\mathrm{ud}}=\frac{\sum_{v\in N(u)}\mathrm{sim}(u,v)\cdot r_{\mathrm{vd}}+\alpha S_{d}}{\sum_{v\in N(u)}|\mathrm{sim}(u,v)|+\alpha }$$

Where *α* ∈ [0,1] is a hyperparameter used to balance the weight between historical ratings and the medical expert’s precise profile score.

Finally, for each health advice seeker, medical experts are ranked according to their predicted ratings ${\hat{r}}_{\mathrm{ud}}$ , and the top *k* experts are selected for the recommendation list. These personalized recommendation results are then presented to the advice seekers to assist them in selecting suitable medical experts that meet their health consultation needs.

## 4 Empirical analysis

### 4.1 Data collection and preprocessing

“Haodf.com,” as a leading domestic online medical platform, features highly structured data with extensive content displayed in web format, making it amenable to collection. Therefore, this study uses this platform as a representative case for empirical analysis.

1) *Data collection*. A total of data on 7,872 medical experts, 533,243 consultation communication records, and 254,402 health consultation evaluation records were collected. The dataset encompasses experts’ basic information, areas of specialization, historical consultation details, and health advice seeker evaluations.
2) *Data preprocessing*. First, the cleaned review texts were tokenized using the “jieba” word segmentation tool. To enhance segmentation accuracy, a professional medical terminology database was integrated into the lexicon using the Open Medical and Health Alliance (OMAHA) knowledge graph platform. Finally, stop words were removed from each review based on the Harbin Institute of Technology Chinese stop word list.

For the rating data, this study further performed user-level mean-centering before calculating user similarity. Specifically, for each health advice seeker (u), we first calculated the average rating $\bar{r_{i}}$ over all medical experts rated by this user. Then, each observed rating *r_i,d_* was transformed into a mean-centered rating:


$$\tilde{r_{i}}=r_{i,d}-\bar{r_{i}}$$

This preprocessing step was used to reduce the bias caused by differences in rating scales among health advice seekers. The cosine similarity calculated based on mean-centered ratings is mathematically equivalent to the Pearson correlation coefficient when it is computed over the set of medical experts commonly rated by two users. Based on this theoretical equivalence, the subsequent collaborative filtering stage adopts this mean-centered formulation for computing user similarities. Therefore, this approach can more accurately capture preference similarity among health advice seekers.

### 4.2 Experimental setup and evaluation metrics

In this experiment, 80% of the entire dataset was randomly selected as the training set, and the remaining 20% was used as the test set. The model was implemented in the following environment: Python = 3.8, PyTorch = 2.0.1.

For the recommendation task, the loss function employed was Mean Squared Error (MSE), and the optimization algorithm used was Adam. A subset of 20% from the training set was designated as the validation set for hyperparameter tuning. The learning rate was set to 0.001, the default setting for Adam, and the batch size was set to 32. Early stopping was applied to prevent overfitting, where training terminates when the validation loss does not decrease for 10 consecutive epochs. The key hyperparameters specific to the RMPE model were optimized via grid search on the validation set. The coefficient *α*, which balances historical ratings and profile scores, was searched over {0.1,0.3,0.5,0.7,0.9}. The coefficient *γ*, which controls the adjustment rate of feature weights, was searched over {0.01,0.05,0.10,0.20}. The coefficient *β*, which balances the SERVQUAL-based structured profile score and the BERT-based semantic score, was searched over {0.10,0.15,0.20,0.25,0.30,0.35,0.40,0.45,0.50}. The optimal configuration was determined as *α* = 0.3, *γ* = 0.05, and *β* = 0.2. The MLP used to map *F_j_* to a semantic score was configured with a 768–128–32-1 architecture, with ReLU activation applied in the hidden layers. Other parameters retained their default values. After training, the model was evaluated on the test set to measure its recommendation performance. The evaluation metrics included Accuracy, F1-score, and Root Mean Squared Error (RMSE).

### 4.3 Analysis of experimental results

#### 4.3.1 Performance evaluation of the sentiment analysis module

To independently evaluate the performance of the sentiment analysis module, we conducted a comparative experiment on the validation set. The residual connection-based LSTM was compared with standard LSTM, BiLSTM, and CNN. The results are shown in Table 3.

**Table 3:** Performance comparison of sentiment analysis models.

Model	Accuracy (%)	F1-score	RMSE	Training time (min)
Standard LSTM	85.30	0.874	0.267	12
Residual Connection-based LSTM	89.20	0.902	0.245	14
BiLSTM	87.50	0.891	0.254	15
CNN	83.10	0.845	0.280	10

The experimental results show that the residual connection-based LSTM outperforms the other comparison models in both accuracy and F1-score. Its accuracy reaches 89.2%, and its F1-score reaches 0.902. Compared with the standard LSTM, the accuracy improves by 3.9 percentage points, while the RMSE decreases from 0.267 to 0.245. Although the introduction of residual connections slightly increases the training time from 12 min to 14 min, this additional computational cost is acceptable given the performance improvement. The ablation experiment further confirms the contribution of the residual connection. After removing the residual connection, the accuracy decreases from 89.2 to 85.3%. These results indicate that the sentiment analysis module can reliably extract sentiment signals from user reviews and provide useful support for the downstream profile-enhanced recommendation task.

#### 4.3.2 Impact of parameters on recommendation performance

In this experiment, RMPE recommendation models with 3, 5, and 7 hidden layers for BERT feature extraction were trained separately, designated as RMPE-3, RMPE-5, and RMPE-7, to examine the influence of the number of hidden layers on model performance. For all models in the experiment, the number of units in each hidden layer was set to 128, and the sigmoid function was used as the activation function. The results are shown in Table 4 and Figure 5.

**Table 4:** The impact of different hidden layers on model performance.

Model name	Number of hidden layers	Accuracy (%)	Loss value
RMPE-3	3	86.3	0.034
RMPE-5	5	87.2	0.033
RMPE-7	7	88.1	0.030

**Figure 5:**
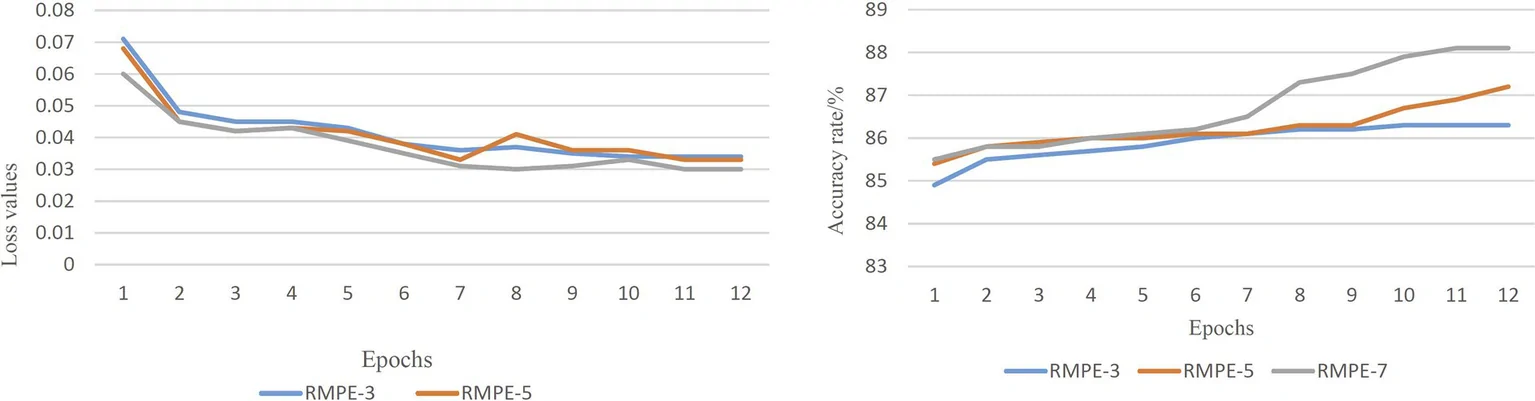
Change curve of RMPE recommendation model metrics under different hidden layers.

On the test set, the RMPE-3 model achieved an accuracy of 86.3% with a loss function value of 0.034. While demonstrating acceptable performance, it did not fully capture the complex relationships within the data. The RMPE-5 model showed improved performance, with accuracy rising to 87.2% and the loss decreasing to 0.033. Compared to RMPE-3, the additional hidden layers enabled the model to better handle non-linear relationships and feature representations in the data. The RMPE-7 model delivered the best performance among the three, attaining an accuracy of 88.1% and further reducing the loss to 0.030. The increased number of hidden layers provided the model with greater capacity to learn complex patterns, resulting in optimal performance in both accuracy and loss metrics.

Based on the experimental results, as the number of hidden layers increased, the model’s performance improved progressively. The RMPE-7 model, in particular, demonstrated the most effective performance during training, outperforming both RMPE-3 and RMPE-5 in terms of the rate of loss function decrease and the final accuracy achieved. This suggests that a deeper network is capable of capturing more complex non-linear relationships within the data, thereby enhancing the model’s overall performance. However, it is important to note that while increasing the number of hidden layers can improve performance, it also incurs greater computational overhead. Therefore, in practical applications, selecting an appropriate model depth requires a balanced consideration of training efficiency and performance. Based on the experimental findings, this study adopts a configuration of 7 hidden layers as the final model setup, and all subsequent mentions of RMPE refer to this 7-layer configuration.

#### 4.3.3 Comparative experiments

To comprehensively evaluate the performance of the RMPE model, we selected a set of representative baselines that cover mainstream recommendation paradigms, including traditional machine learning classifiers, sequence modeling approaches, generative adversarial learning methods, and classical collaborative filtering. These baselines are widely adopted in recommendation system research and provide a reasonable reference for assessing the effectiveness of the proposed profile-enhancement approach.

To ensure a fair comparison, all baseline models used the same training and test sets as RMPE, and the feature inputs were kept consistent. The traditional machine learning models were implemented using the standard functions in the scikit-learn library. The deep learning models were constructed using standard components in the PyTorch framework. The specific parameter settings of the baseline models were as follows. Support Vector Machine (SVM) used the Radial Basis Function (RBF) kernel, and the penalty parameter C was determined through grid search over {0.1, 1, 10}. Logistic Regression (LR) used L2 regularization, and the inverse regularization strength C was also tuned over {0.1, 1, 10}. Random Forest (RF) was configured with 100 decision trees, and the maximum depth was determined as 10 based on validation set performance. For the Recurrent Neural Network (RNN), a single-layer unidirectional LSTM structure was adopted. The hidden dimension was set to 128, which was consistent with the LSTM component in RMPE, and Adam was used as the optimizer. For the Generative Adversarial Network (GAN), both the generator and the discriminator were constructed as three-layer fully connected networks. Their hidden dimensions were set to 128 and 64, respectively. Recommendation ranking was optimized through adversarial training, and the optimizer was kept consistent with RMPE. For Matrix Factorization (MF), the user-expert rating matrix was factorized into low-dimensional user and item latent factor matrices using the SVD algorithm, with the latent factor dimension set to the default value of 100 and the regularization parameter tuned on the validation set. The experimental results are shown in Table 5.

**Table 5:** Comparative experimental results.

Experiment type	Model	Accuracy	F1-score	RMSE
Comparative experiment	RMPE	0.88	0.85	0.12
	Support Vector Machine (SVM)	0.78	0.75	0.18
	Logistic Regression (LR)	0.74	0.72	0.21
	Random Forest (RF)	0.80	0.78	0.16
	Recurrent Neural Network (RNN)	0.83	0.81	0.14
	Generative Adversarial Networks (GAN)	0.84	0.82	0.13
	Matrix Factorization (MF)	0.81	0.79	0.15

The experimental results show that, among the traditional machine learning models, SVM achieved moderate performance but failed to effectively capture the complex relationships in the data. LR performed relatively poorly, indicating its limitations in recommendation tasks. RF achieved better performance than SVM and LR, but it still underperformed the RMPE model. MF, as a classical collaborative filtering approach, achieved competitive performance among the baseline models, outperforming the traditional machine learning classifiers. Among the deep learning baselines, RNN and GAN achieved relatively strong performance, further surpassing MF. However, they still lagged behind RMPE in terms of accuracy and RMSE, and they also involved higher computational complexity. In comparison, RMPE achieved the best results across all evaluation metrics. These findings verify the effectiveness of the profile-enhancement approach.

It is important to note that emerging techniques such as Graph Neural Networks (GNNs) and ensemble learning aimed at enhancing model robustness have also been introduced into recommendation systems and related fields in recent years (29, 30). These methods hold potential for uncovering complex relationships. However, the focus of this study is to validate the effectiveness of the ‘profile enhancement’ approach within a collaborative filtering framework. The baselines selected for the RMPE model are already sufficient to support this core objective-namely, to demonstrate that systematically constructing and integrating dynamic expert profiles can significantly improve the performance of recommendations in online health communities.

#### 4.3.4 Cross-departmental validation

To further examine the robustness of the RMPE model across different clinical contexts, we partitioned the dataset into four specialty subsets according to the consultation domains recorded in the original data: General Internal Medicine, Cardiology, Neurology, and Gastroenterology. The model was evaluated separately on each subset using the same experimental settings as the main experiment. The results are presented in Table 6.

**Table 6:** Performance of RMPE across different clinical departments.

Department	Accuracy	F1-score	RMSE
General internal medicine	0.876	0.858	0.112
Cardiology	0.883	0.851	0.119
Neurology	0.875	0.821	0.128
Gastroenterology	0.868	0.837	0.132

The results demonstrate that RMPE maintains consistent performance across all four clinical departments, with accuracy ranging from 0.868 to 0.883, F1-score ranging from 0.821 to 0.858, and RMSE ranging from 0.112 to 0.132. This cross-departmental consistency confirms that the model is not limited to a single scenario but exhibits strong adaptability across diverse clinical settings and application contexts.

### 4.4 Validation of recommendation results

To objectively evaluate the performance of the RMPE recommendation model in real-world scenarios, this study conducted a retrospective validation of the model’s recommendation effectiveness using real data crawled from the “Haodf.com” platform. The core method involved generating recommendation lists based on health advice seekers’ historical behavior and comparing them with the advice seekers’ actual final choices. The recommendation accuracy was quantified by calculating the Return Probability, defined as: Return Probability (Top-N) = (Number of advice seekers whose final chosen expert appears in the Top-N recommendation list)/Total number of advice seekers.

#### 4.4.1 Selection of health advice seekers for testing and the validation benchmark

This study randomly selected 300 health advice seekers with unambiguous final consultation records from the crawled dataset, covering the four consultation domains of General Internal Medicine, Neurology, Gastroenterology, and Cardiovascular Medicine. These advice seekers served as the test set. The medical expert with whom an advice seeker actually completed a consultation, as recorded in the crawled data, was explicitly defined as that advice seeker’s final choice expert. This serves as the ground truth benchmark for validating the model’s recommendation effectiveness.

#### 4.4.2 Generating personalized recommendation lists

For each test health advice seeker, their review history data from the crawled dataset-specifically, the data prior to their final consultation record-were used as input for the RMPE model. The RMPE model independently generated a personalized expert recommendation list for each advice seeker, ranked from highest to lowest predicted interest, with a list length of *N*.

#### 4.4.3 Effect comparison and hit rate calculation

The model’s recommendation results were compared against the ground truth benchmark to calculate the return probability, that is, the proportion of advice seekers whose “finally selected medical expert” appeared in their Top-N recommendation list.

Following the above validation process, this study calculated the return probabilities across four medical departments for different recommendation list lengths Top-10, Top-20, Top-30. The specific results are presented in Figure 6.

**Figure 6:**
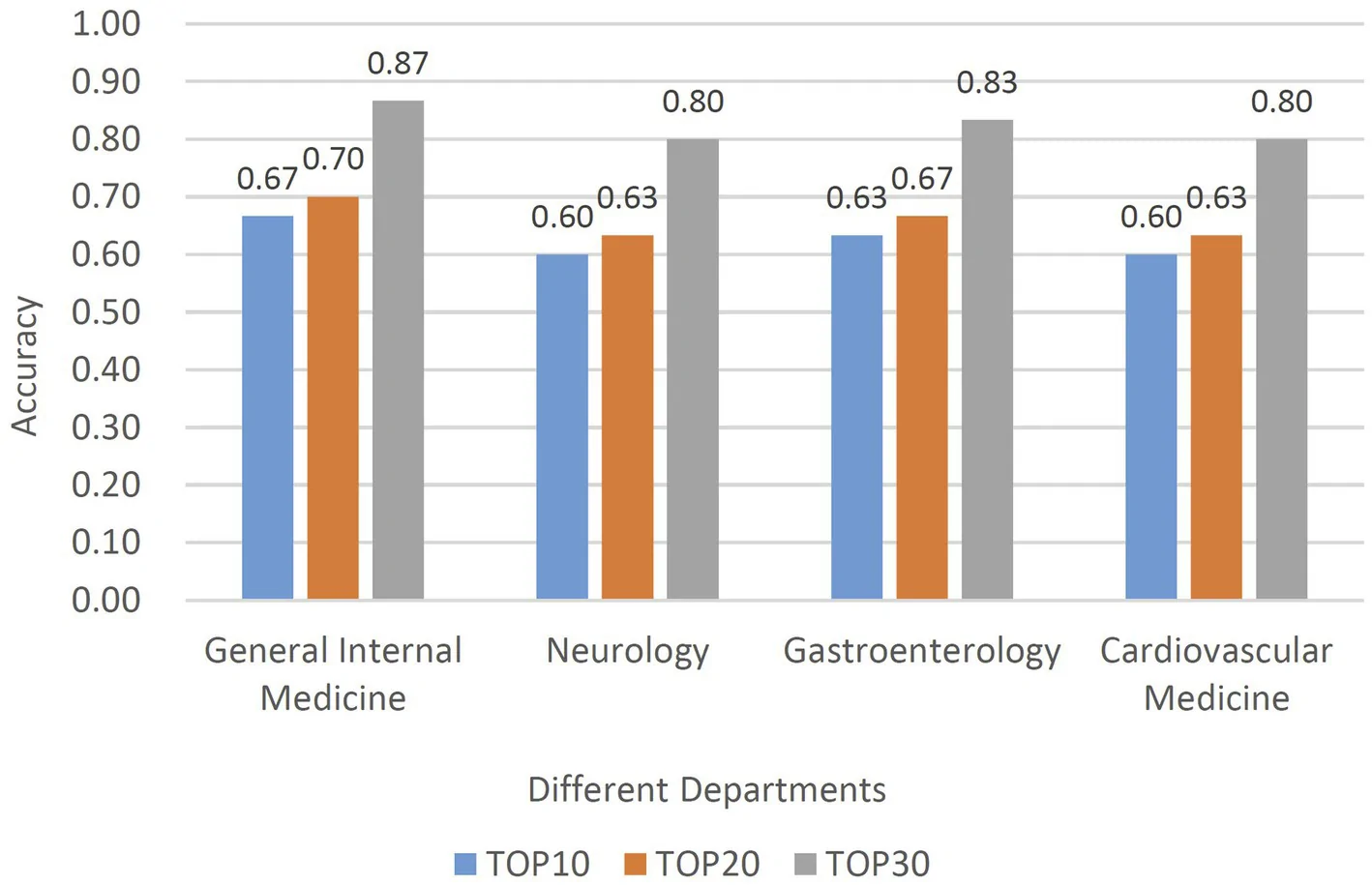
Comparison of return probability results from medical expert recommendation sets.

The experimental results indicate that as the recommendation list length N increases, the return probabilities for all departments show a significant upward trend. Specifically, for the Top-10 list, the return probabilities across the four departments range between 0.60 and 0.67. When the list expands to Top-20, the hit rate range improves to 0.63–0.70. In the Top-30 list, the return probabilities further climb to a high level of 0.80–0.87. This pattern demonstrates that the RMPE model can effectively rank the medical experts ultimately chosen by advice seekers near the top of the recommendation list. Although the model’s coverage of potential choices is less comprehensive in shorter lists such as Top-10, its ability to capture advice seekers’ genuine preferences becomes fully apparent as the list expands. In the Top-30 list, it successfully covers over 80% of the advice seekers’ actual choices. These results empirically confirm that the RMPE recommendation model, enhanced by integrated profiling, can effectively improve the accuracy of medical expert-health advice seeker matching in practice, demonstrating sound theoretical and practical value.

## 5 Implications for online health communities

Integrating Service-Dominant Logic and Information Ecology Theory, this study constructs a profile-enhanced personalized medical expert recommendation model for online health communities, namely RMPE. Rigorous empirical analysis demonstrates that, by synthesizing medical expert–health advice seeker interaction resources, constructing multidimensional dynamic expert profiles, and incorporating user sentiment feedback, the model significantly improves recommendation accuracy. This validates the effectiveness of the profile enhancement pathway in optimizing medical expert-health advice seeker matching.

The primary theoretical contribution of this research lies in providing a computable framework for analyzing value co-creation, tailored to the platform ecosystem of online health communities. By operationalizing Service-Dominant Logic into dynamic profiles that integrate SERVQUAL dimensions and leveraging sentiment analysis to quantify users’ value perceptions, the RMPE model empowers platform managers to systematically measure and analyze the co-created value emerging from expert-advice seeker interactions within health communities. This approach transcends traditional recommendation systems that rely on static attributes or textual similarity, offering a new research perspective for a deeper understanding of the interactive nature and ecological health of professional service platforms in the health domain.

At the practical level, this research offers practical implications for governing and optimizing online health communities. The RMPE model provides community managers with a transparent tool for optimizing the core matching mechanism; the multi-dimensional profiles it generates can serve as a real-time diagnostic dashboard for service quality, helping identify top contributors and key areas for improvement. More importantly, by quantifying and emphasizing soft service dimensions such as empathy and responsiveness, the model empirically demonstrates that cultivating a trustworthy and supportive community culture plays a decisive role in enhancing user retention and platform sustainability. Thus, this study not only improves matching efficiency but also provides a data-driven decision-making paradigm for building a healthier and more sustainable internet-based health community ecosystem.

## 6 Discussion and future work

This study addresses the inefficiency of matching between medical experts and health advice seekers in online health communities by proposing the RMPE model. This model is grounded in Service-Dominant Logic and Information Ecology Theory. It integrates BERTopic and an enhanced SERVQUAL framework to construct dynamic expert profiles, employs a residual-connected LSTM for sentiment analysis, and incorporates a collaborative filtering mechanism to achieve personalized matching. Empirical results demonstrate that RMPE achieves a recommendation accuracy of 88.1%, significantly outperforming traditional baselines, and successfully identifies over 80% of users’ final choices within the Top-30 recommendation list. Cross-departmental validation on four specialty subsets further confirms the model’s robustness, with consistent performance across General Internal Medicine, Cardiology, Neurology, and Gastroenterology. These findings not only validate the technical effectiveness of the profile enhancement pathway but also provide a computable framework for analyzing value co-creation in online health communities.

Although this study has achieved certain progress, limitations remain. The empirical analysis was conducted solely on the Haodf.com platform. In practice, applying the RMPE model to a new platform would require careful adaptation to platform-specific data characteristics. User-generated review texts vary across platforms in terms of linguistic style, domain terminology, and length distribution, which may affect the performance of both BERTopic-based topic extraction and LSTM-based sentiment classification. Rating mechanisms also differ across platforms. Some platforms use five-point or ten-point scales, while others rely solely on textual feedback without explicit ratings. When the data distribution shifts, hyperparameters such as the number of topics in BERTopic, the balancing coefficient *α*, and the adjustment rate *γ* in collaborative filtering may need to be re-tuned, as the topic structure and rating sparsity patterns are likely to differ across platforms. Addressing these factors is essential for successfully deploying the model in new settings. We will systematically conduct cross-platform validation in our future work, which involves multiple stages such as multi-source data collection and cleaning, alignment and standardization of data structures across platforms, and re-tuning of model hyperparameters on new data, to further examine the performance of the RMPE model across different platforms. In addition, the current model uses health advice seekers’ emotional feedback in the form of a global sentiment score. This is a design choice driven by the collaborative filtering mechanism to ensure that ratings from different users are comparable on a unified scale. To further improve the degree of personalization in recommendations, inspired by recent advances in personalized recommendation, we plan to explore aspect-level sentiment analysis that captures users’ differentiated perceptions across service quality dimensions, thereby integrating personalized preferences into the existing recommendation framework in our future work.

## Data Availability

The original contributions presented in the study are included in the article/supplementary material, further inquiries can be directed to the corresponding author.
